# MSACompro: protein multiple sequence alignment using predicted secondary structure, solvent accessibility, and residue-residue contacts

**DOI:** 10.1186/1471-2105-12-472

**Published:** 2011-12-14

**Authors:** Xin Deng, Jianlin Cheng

**Affiliations:** 1 Department of Computer Science, University of Missouri-Columbia, Columbia, MO 65211, USA; 2 Informatics Institute, University of Missouri-Columbia, Columbia, MO 65211, USA; 3 C. Bond Life Science Center, University of Missouri-Columbia, Columbia, MO 65211, USA

## Abstract

**Background:**

Multiple Sequence Alignment (MSA) is a basic tool for bioinformatics research and analysis. It has been used essentially in almost all bioinformatics tasks such as protein structure modeling, gene and protein function prediction, DNA motif recognition, and phylogenetic analysis. Therefore, improving the accuracy of multiple sequence alignment is important for advancing many bioinformatics fields.

**Results:**

We designed and developed a new method, MSACompro, to synergistically incorporate predicted secondary structure, relative solvent accessibility, and residue-residue contact information into the currently most accurate posterior probability-based MSA methods to improve the accuracy of multiple sequence alignments. The method is different from the multiple sequence alignment methods (e.g. 3D-Coffee) that use the tertiary structure information of some sequences since the structural information of our method is fully predicted from sequences. To the best of our knowledge, applying predicted relative solvent accessibility and contact map to multiple sequence alignment is novel. The rigorous benchmarking of our method to the standard benchmarks (i.e. BAliBASE, SABmark and OXBENCH) clearly demonstrated that incorporating predicted protein structural information improves the multiple sequence alignment accuracy over the leading multiple protein sequence alignment tools without using this information, such as MSAProbs, ProbCons, Probalign, T-coffee, MAFFT and MUSCLE. And the performance of the method is comparable to the state-of-the-art method PROMALS of using structural features and additional homologous sequences by slightly lower scores.

**Conclusion:**

MSACompro is an efficient and reliable multiple protein sequence alignment tool that can effectively incorporate predicted protein structural information into multiple sequence alignment. The software is available at http://sysbio.rnet.missouri.edu/multicom_toolbox/.

## Background

Aligning multiple evolutionarily related protein sequences is a fundamental technique for studying protein function, structure, and evolution. Multiple sequence alignment methods are often an essential component for solving challenging bioinformatics problems such as protein function prediction, protein homology identification, protein structure prediction, protein interaction study, mutagenesis analysis, and phylogenetic tree construction. During the last thirty years or so, a number of methods and tools have been developed for multiple sequence alignment, which have made fundamental contributions to the development of the bioinformatics field.

State of the art multiple sequence alignment methods adapt some popular techniques to improve alignment accuracy, such as iterative alignment [1], progressive alignment [2], alignment based on profile hidden Markov models [3], and posterior alignment probability transformation [4,5]. Some alignment methods, such as 3D-Coffee [6] and PROMALS3D [7], use 3D structure information to improve multiple sequence alignment, which cannot be applied to the majority of protein sequences without tertiary structures. In order to overcome this problem, we have developed a method to incorporate secondary structure, relative solvent accessibility, and contact map information predicted from protein sequences into multiple sequence alignment. Predicted secondary structure information has been used to improve pairwise sequence alignment [8,9], but few attempts had been made to use predicted secondary structure information in multiple sequence alignment [10-15]. To the best of our knowledge, applying predicted relative solvent accessibility and residue-residue contact map to multiple sequence alignment is novel.

In order to use the predicted structural information to advance the state of the art of multiple sequence alignment, we first compared the existing multiple sequence alignment tools [16,4,5-37] on the standard benchmark data sets such as BAliBASE [38], SABmark [39] and OXBENCH [40], which showed that MAFFT [30], T-coffee [31], MSAProbs [4], and ProbCons [5] yielded the best performance. Then we developed MSACompro, a new multiple sequence alignment method, which effectively utilizes predicted secondary structure, relative solvent accessibility, and residue-residue contact map together with posterior alignment probabilities produced by both pair hidden Markov models and partition function as in MSAProbs [4]. The assessment results of MSACompro compared to the benchmark data sets from BAliBASE, SABmark and OXBENCH showed that incorporating predicted structural information has improved the accuracy of multiple sequence alignment over most existing tools without using structural features and sometimes the improvement is substantial.

## Method

Following the general scheme in MSAProbs [4], MSACompro has five main steps: (1) compute the pairwise posterior alignment probability matrices based on both pair-HMM and partition function, considering the similarity in amino acids, secondary structure, and relative solvent accessibility; (2) generate the pairwise distance matrix from both the pairwise posterior probability matrices constructed in the first step and the pairwise contact map similarity matrices; (3) construct a guide tree based on pairwise distance matrix, and calculate sequence weights; (4) transform all the pairwise posterior matrices by a weighting scheme; (5) perform a progressive alignment by computing the profile-profile alignment from the probability matrices of all sequence pairs, and then an iterative alignment to refine the results from progressive alignment. Our method is different from MSAProbs in that it adds secondary structure and solvent accessibility information to the calculation of the posterior residue-residue alignment probabilities and computes the pairwise distance matrix with the help of predicted residue-residue contact information.

### Construction of pairwise posterior probability matrices based on amino acid sequence, secondary structure and solvent accessibility information

For two protein sequences X and Y in a sequence group S to be aligned, we denote *X *= (*x*_1_, *x*_2_,......,*x*_*n*__1_), *Y *= (*y*_1_, *y*_2_,......,*y*_*n*__2_), where *x*_1_, *x*_2_,......, *x*_*n*__1 _and *y*_1_, *y*_2_,......,*y*_*n*__2 _are lists of the residues in X and Y, respectively. *n*_1 _is the length of sequence X, and *n*_2 _is the length of sequence Y. Suppose *x*_*i *_is the i-th amino acid in sequence X, and *y*_*j *_is the j-th amino acid in sequence Y. We let *aln *denote a global alignment between X and Y, *ALN *the set of of all the possible global alignments of X and Y, and aln* ∈ *ALN *the true pairwise alignment of X and Y. The posterior probability that the i-th residue in X (*x*_*i*_) is aligned to the j-th residue (*y*_*j*_) in Y in aln* is defined as:

(1) $$\begin{array}{l} p(x_{i}\sim y_{j}\in aln^{*}|X,Y)=\\ \sum_{aln\in ALN}P(aln|X,Y)I\{x_{i}\sim y_{j}\in aln\} \end{array}$$

$$\left(1\leq x_{i}\leq n_{1},1\leq y_{j}\leq n2\right)$$

$$I\{x_{i}\sim y_{j}\in aln\}=\left\{ \begin{array}{l} 1,if(x_{i}\sim y_{j}\in aln)true\\ o,otherwise \end{array}\right.$$

*P*(*aln *| *X*, *Y*) denotes the probability that *aln *is the true alignment aln*: Thus, the posterior probability *n*_1 _× *n*_2 _matrix *P*_*XY *_is a collection of all the values *p*(*x*_*i *_~ *y*_*j *_∈ aln* | *X*, *Y*) (*p*(*x*_*i *_~ *y*_*j*_) for short) for 1 ≤ *x*_*i *_≤ *n*_1_, 1 ≤ *y*_*j *_≤ *n*2. The calculation process of the pairwise posterior probability matrix is described as follows.

As in MSAProbs, two different methods (a pair hidden Markov model and a partition function) are used to compute the pairwise posterior probability matrices ($P_{XY}^{1}$ and $P_{XY}^{2}$), respectively. The first kind of pairwise probability matrix $P_{XY}^{1}$ is calculated by a partition function (*F*) of alignments based on dynamic programming. *F*(*i*, *j*) denotes the probability of all partial global alignments of X and Y ending at position (i, j). *F*_*M *_(*i*, *j*) is the probability of all partial global alignments with *x*_*i *_aligned to *y*_*j*_, *F*_*y*_(*i*, *j*), is the probability of all partial global alignments with *y*_*j *_aligned to a gap, and *F*_*X*_(*i*, *j*) is the probability of all partial global alignments with *x*_*i *_aligned to a gap. Accordingly, the partition function can be calculated recursively as follows:

$$F_{M}\left(i,j\right)=F\left(i-1,j-1\right)e^{W_{1}\beta s\left(x_{i},y_{j}\right)+W_{2}SS\left(ss\left(x_{i}\right),ss\left(y_{j}\right)\right)+W_{3}SA\left(sa\left(x_{i}\right),sa\left(y_{j}\right)\right)}$$

(2) $$F_{Y}\left(i,j\right)=F_{M}\left(i,j-1\right)e^{\beta gap}+F_{Y}\left(i,j-1\right)e^{\beta ext}$$

$$F_{X}\left(i,j\right)=F_{M}\left(i-1,j\right)e^{\beta gap}+F_{X}\left(i-1,j\right)e^{\beta ext}$$

$$F\left(i,j\right)=F_{M}\left(i,j\right)+F_{Y}\left(i,j\right)+F_{X}\left(i,j\right)$$

Subject to the constraint *W*_1 _+ *W*_2 _+ *W*_3 _= 1.

In the formula above, *s*(*x*_*i*_, *y*_*j*_) is the amino acid similarity score between *x*_*i *_and *y*_*j*_. One element of the substitution matrix s, *SS*(*ss*(*x*_*i*_), *ss*(*y*_j_)) is the similarity score between the secondary structure (*ss*(*x*_*i*_)) of residue *x*_*i *_in protein X and that of residue *y*_*j *_in protein Y according to the secondary structure similarity matrix SS, *SA*(*sa*(*x*_*i*_), *sa*(*y*_*j*_)) is the similarity score between the relative solvent accessibility (*sa*(*x*_*i*_)) of residue *x*_*i *_in protein X and that of residue *y*_*j *_in protein Y according to the solvent accessibility similarity matrix SA. *W*_1_, *W*_2_, *W*_3 _are weights used to control the influence of the amino acid substitution score, secondary structure similarity score, and solvent accessibility similarity score. The secondary structure and solvent accessibility can be automatically predicted by SSpro/ACCpro [41] (http://sysbio.rnet.missouri.edu/multicom_toolbox/) using a multi-threading technique implemented in MSACompro, or alternatively be provided by a user. The values of the three weights are set to 0.4, 0.5, and 0.1 by default, and can be adjusted by users. The ensembles of bidirectional recurrent neural network architectures in ACCpro are used to discriminate between two different states of relative solvent accessibility, higher or lower than the accessibility cutoff - 25% of the total surface area of a residue [42], corresponding to e or b. As in MSAprobs, *β *is a parameter measuring the deviation between suboptimal and optimal alignments, *gap*(*gap *≤ 0) is the gap open penalty, and *ext*(*ext *≤ 0) is the gap extension penalty.

We used the Gonnet 160 matrix as a substitution matrix to generate the similarity scores between two amino acids in proteins [43]. The 3 × 3 secondary structure similarity matrix SS contains the similarity scores of three kinds of secondary structures (E, H, C) as follows:

$$SS=\left[\begin{array}{c} 100\\ 010\\ 001 \end{array}\right]$$

, where two identical secondary structures receive a score of 1 and different ones receive a score of 0.

The 2 × 2 solvent accessibility similarity matrix SA contains the similarity scores of two kinds of relative solvent accessibilities (e, b) as follows:

$$SA=\left[\begin{array}{c} 10\\ 01 \end{array}\right]$$

, where two identical solvent accessibilities receive a score of 1 and different ones receive a 0. It is worth noting that we used the simple identity scoring matrix for secondary structure and solvent accessibility here. Employing more advance scoring matrices defined in [44] may lead to further improvement. Each posterior residue-residue alignment probability element in the first kind of posterior probability matrix ($P_{XY}^{1}$) can be calculated from the partition function as:

(3) $$\begin{array}{l} p^{1}(x_{i}\sim y_{j})=\frac{F_{M}(i-1,j-1)F_{M}^{'}(i+1,j+1)}{F}•\\ e^{W_{1}\beta s(x_{i},y_{j})+W_{2}SS(ss(x_{i}),ss(y_{j}))+W_{3}SA(sa(x_{i}),sa(y_{j}))} \end{array}$$

, where $F_{M}\prime \left(i,j\right)$ denotes the partition function of all the reverse alignments starting from the position (n_1_, n_2_) till position (*i*, *j*) with *x*_*i *_aligned to *y*_*j*_.

As in MSAProbs, the second kind of pairwise probability matrix $P_{XY}^{2}$ is calculated by a pair hidden Markov model (HMM) combining both Forward and Backward algorithm [4,5,45]. The pairwise probabilities can be generated under the guidance of pair HMM involving state emissions and transitions. $P_{XY}^{2}$ is only derived from protein sequences without using secondary structure and solvent accessibility, which is different from PROMALS [15] that lets HMM emit both amino acids and secondary structure alphabets.

The final posterior probability matrix *P*_*XY *_is calculated as the root mean square of the corresponding values in $P_{XY}^{1}$ and $P_{XY}^{2}$ as follows.

(4) $$p(x_{i}\sim y_{j})=\sqrt{\frac{p^{1}{\left(x_{i}\sim y_{j}\right)}^{2}+p^{2}{\left(x_{i}\sim y_{j}\right)}^{2}}{2}}$$

where *p*^1^(*x*_*i *_~ *y*_*i*_) and *p*^2^(*x*_*i *_~ *y*_*i*_) denote a posterior probability element in two kinds of posterior probability matrices ($P_{XY}^{1}$ and $P_{XY}^{2}$), respectively.

### Construction of pairwise distance matrices based on pairwise posterior probabilities and pairwise contact map scores

The posterior probability matrix *P*_*XY *_is used as a scoring function to generate a pairwise global alignment between sequences X and Y. The optimal global alignment score Opt(X,Y) of the global alignment is computed according to an optimal sub-alignment score matrix AS. The optimal sub-alignment score AS(i, j) denotes the score of the optimal sub-alignment ending at residues *i *and *j *in X and Y. The AS matrix is recursively calculated as:

(5) $$AS(i,j)=\max\left\{ \begin{array}{l} AS(i-1,j-1)+P_{XY}(x_{i}\sim y_{j})\\ AS(i-1,j)\\ AS(i,j-1) \end{array}\right.$$

*AS *(*n*_1_, *n*_2_) is the optimal score of the full global alignment between X and Y, which is denoted as *Optscore*(*X*,*Y*).

In addition to the optimal alignment score, we introduce a contact map score, *CMscore*(*X*, *Y*), for the optimal pairwise alignment of X and Y, assuming that the spatially neighboring residues of two aligned residues should have a higher tendency to be aligned together. *CMscore*(*X*, *Y*) is calculated from the contact map correlation score matrix *CMap*_*XY *_based on the residue-residue contact map matrices *CMap*_*X *_and *CMap*_*Y *_of X and Y.

Assuming the optimal global alignment of X and Y is represented as,

$$\begin{array}{c} x_{1}x_{2}.......-x_{m}......x_{p}......x_{n1}\\ y_{1}-......y_{k}y_{k+1}.....-......y_{n2} \end{array}$$

we can generate a new alignment after removing the pairs containing gaps:

$$\begin{array}{c} x_{1}.......x_{m}............x_{n1}\\ y_{1}......y_{k+1}...........y_{n2} \end{array}$$

, which can be denoted as

$$\begin{array}{c} x_{1}^{\prime }x_{2}^{\prime }............x_{n}^{\prime }\\ y_{1}^{\prime }y_{2}^{\prime }...........y_{n}^{\prime } \end{array}$$

, where *n *is the length of the new alignment without gaps

From this alignment, we can construct two contact map matrices, *CMap*_*X *_and *CMap*_*Y*_, shown below:

(6) $$CMap_{X}=\left[\begin{array}{c} x_{11}^{\prime }x_{12}^{\prime }......x_{1n}^{\prime }\\ x_{21}^{\prime }x_{22}^{\prime }......x_{2n}^{\prime }\\ ...................\\ ...................\\ x_{n1}^{\prime }x_{n2}^{\prime }......x_{nn}^{\prime } \end{array}\right]$$

$$CMap_{Y}=\left[\begin{array}{c} y_{11}^{\prime }y_{12}^{\prime }......y_{1n}^{\prime }\\ y_{21}^{\prime }y_{22}^{\prime }......y_{2n}^{\prime }\\ ...................\\ ...................\\ y_{n1}^{\prime }y_{n2}^{\prime }......y_{nn}^{\prime } \end{array}\right]$$

$x_{ij}^{\prime }$ is the contact probability score between amino acid $x_{i}^{\prime }$ and $x_{j}^{\prime }$ in protein sequence X, and $y_{ij}^{\prime }$ is the contact probability score between amino acid $y_{i}^{'}$ and $y_{j}^{'}$ in protein sequence Y. The residue-residue contact probabilities are predicted from the sequence by NNcon [46] (http://sysbio.rnet.missouri.edu/multicom_toolbox/). The contact map correlation score matrix *CMap*_*XY *_is calculated as the multiplication of *CMap*_*X *_and *CMap*_*Y*_:

(7) $$\begin{array}{c} CMap_{XY}=CMap_{X}\times CMap_{Y}\\ =\left[\begin{array}{c} xy_{11}^{\prime }xy_{12}^{\prime }....xy_{1n}^{\prime }\\ xy_{21}^{\prime }xy_{22}^{\prime }....xy_{2n}^{\prime }\\ ......................\\ ......................\\ xy_{n1}^{\prime }xy_{n2}^{\prime }....xy_{nn}^{\prime } \end{array}\right] \end{array}$$

$xy_{ii}^{\prime }$ is the contact map score for an aligned residue pair (amino acid $x_{i}^{\prime }$ in protein X and amino acid $y_{i}^{'}$ in protein Y). The contact map score for the global alignment of two sequences X and Y is calculated as

(8) $$\begin{array}{c} CMscore\left(X,Y\right)=\frac{1}{n^{2}}\overset{n}{\underset{i=1}{\sum }}CMap_{XY}\left(i,i\right)\\ =\frac{1}{n^{2}}\overset{n}{\underset{i=1}{\sum }}xy_{ii}^{\prime }=\frac{1}{n^{2}}\overset{n}{\underset{i=1}{\sum }}\overset{n}{\underset{j=1}{\sum }}x_{ij}^{\prime }y_{ji}^{\prime } \end{array}$$

In practice, we only need to calculate the diagonal values in *CMap*_*XY*_.

Finally, we define the pairwise distance between sequences X and Y as

(9) $$d\left(X,Y\right)=1-\frac{W_{4}Optscore\left(X,Y\right)}{\min\left\{n_{1},n_{2}\right\}}-W_{5}CMscore\left(X,Y\right)$$

, where *W*_4 _+ *W*_5 _= 1. The weights *W*_4 _and *W*_5 _are used to control the influence of sequences X and Y.

### Construction of guide tree and transformation of posterior probability

Akin to MSAProbs [4], a guide tree is constructed by the UPGMA method that uses the linear combinatorial strategy [47]. The distance between a new cluster Z formed by merging clusters X and Y, and another cluster W is calculated as (10):

(10) $$d\left(W,Z\right)=\frac{d\left(W,X\right)\times Num\left(X\right)+d\left(W,Y\right)\times Num\left(Y\right)}{Num\left(X\right)+Num\left(Y\right)}$$

In which Num(X) is the number of leafs in cluster X.

After the guide tree is constructed, sequences are weighted according to the schemes inferred in [4].

To reduce the bias of sampling similar sequences, we use a weighted scheme to transform the former posterior probability as

(11) $$P_{XY}^{\prime }=\frac{1}{wN}\left(\left(w_{X}+w_{Y}\right)P_{XY}+\underset{Z\in S,Z\neq X,Y}{\sum }w_{z}P_{XZ}P_{ZY}\right)$$

*w*_*X *_and *w*_*Y *_are, respectively, the weight of sequences X and Y, *w*_*Z *_is the weight of a sequence Z other than X or Y in the given group of sequences, and wN is the sum of sequence weights in dataset S.

### Combination of progressive and iterative alignment

We first use the guide tree to generate a multiple sequence alignment by progressively aligning two clusters of the most similar sequences together. As in MSAProbs [4], we also apply a weighted profile-profile alignment to align two clusters of sequences. The sequence weights are the same as in the previous step. The posterior alignment probability matrix of two clusters/profiles is averaged from the probability matrices of all sequence pairs (X, Y), where x and y are from the two different clusters. Formula (5) used to generate the global profile-profile alignment is based on the posterior alignment probability matrices of the profiles. In order to further improve the alignment accuracy, we then use a randomized iterative alignment to refine the initial alignment. This randomized iterative refinement randomly partitions the given sequence group S into two separate groups, and performs a profile-profile alignment of the two groups. The iterative refinement can be completed after 10 iterations by default, or a fixed number of iterations set by users. Generally speaking, the final progressive alignment orders sequences along the guide tree from closely related to distantly related. To improve the alignment accuracy, a final iterative alignment is applied to refine the results from progressive alignment. In addition, a multi-thread technology based on OpenMP is also used to improve the efficiency of the program [48].

## Results and discussion

### Evaluation of MSACompro and other tools on the standard benchmarks

We tested MSACompro in comparison to three benchmarks: BAliBASE, SABmark and OXBENCH, and evaluated the alignment results in terms of sum-of-pairs (SP) score and true column (TC) score. The SP score is the number of correctly aligned pairs of residue in the test alignment divided by the total number of aligned pairs of residues in core blocks of the reference alignment [49]. The TC score is the number of correctly aligned columns in the test alignment divided by the total number of aligned columns in core blocks of the reference alignment [49]. We used the application bali_score provided by BAliBASE 3.0 to calculate these scores. We compared MSACompro to 11 other MSA tools which do not have access to the structural information, including ClustalW 2.0.12, DIALIGN-TX 1.0.2 [27], FSA 1.15.5, MAFFT 6.818, MSAProbs 0.9.4, MUSCLE 3.8.31, Opal 0.2.0, POA 2, Probalign 1.3, Probcons and T-coffee 8.93. It is worth noting that a fair comparison between our method with these multiple sequence alignment methods without using structural features is not possible because these methods use less input information. So, the goal of comparison is to present the idea that structural information-based alignment may contain valuable information that is not available in sequence-based multiple sequence alignments and can therefore be a supplement to sequence-based alignments. And to make the evaluation more fair and comprehensive, we also compared MSACompro with four tools which use structural information, including MUMMALS 1.01 [14], PROMALS [15] and PROMALS3D [7].

To understand how various parameters of MSACompro affect alignment accuracy, some experiments were carried out to evaluate these variants based on two algorithm changes: (1) combining amino acids, secondary structure, and relative solvent accessibility information into the partition function calculation using respective weights for each of them; (2) computing the pairwise distance from both the pairwise posterior probability matrices and the pairwise contact map similarity matrices by introducing the weight wc for contact map information. To optimize the parameters, we used BAliBASE 3.0 data sets as training sets, and SABmark 1.65 and OXBENCH data sets as testing sets. Firstly, we focused on the effect of secondary structure and solvent accessibility information by testing different values of weight w_1 _for amino acid similarity and weight w_2 _for secondary structure information on BAliBASE 3.0 data sets. MSACompro worked wholly the best if the weight w_1 _for amino acid similarity and the weight w_2 _for secondary structure information were 0.4 and 0.5, respectively. Since the sum of w_1_, w_2 _and w_c _is 1, we can deduce that w_c _is 0.1 if w_1 _and w_2 _are 0.4 and 0.5. Then we focused on the effect of residue-residue contact map information under two different scenarios: using secondary structure and relevant solvent accessibility information by keeping the w_1_, w_2_, and w_3 _at their optimum values (0.4, 0.5, 0.1), or excluding that information by setting both w_2 _and w_3 _as 0. Evaluation results on BAliBASE 3.0 database were found to improve the most when w_c _is 0.9 by integrating both secondary structure and relevant solvent accessibility information. Additionally, to avoid over-fitting, we tested MSACompro against SABmark 1.65 and OXBENCH data sets using this set of parameters independently, and found that a significant improvement was also gained in comparison to other leading protein multiple sequence alignment tools. More details can be found in the next section, "A comprehensive study on the effect of predicted structural information on the alignment accuracy". Consequently, the weights w_1_, w_2_, w_3 _and w_c _are respectively set at 0.4, 0.5, 0.1 and 0.9 in MSACompro by default. All other tools were also evaluated under default parameters.

Firstly, we evaluated these methods on BAliBASE [16] - the most widely used multiple sequence alignment benchmark. The latest version, BAliBASE 3.0, contains 218 reference alignments, which are distributed into five reference sets. Reference set 1 is a set of equal-distant sequences, which are organized into two reference subsets, RV11 and RV12. RV11 contains sequences sharing >20% identity and RV12 contains sequences sharing 20% to 40% identity. Reference set 2 contains families with >40% identity and a significantly divergent orphan sequence that shares <20% identity with the rest of the family members. Reference set 3 contains families with >40% identity that share <20% identity between each two different sub-families. Reference set 4 is a set of sequences with large N/C-terminal extensions. Reference set 5 is a set of sequences with large internal insertions. Tables 1, 2, and 3 report the mean SP scores and TC scores of MSACompro and the tools without using structural information for the six subsets and the whole database. All the scores in the tables are multiplied by 100, and the highest scores in each column are marked in bold. The results show that MSACompro received the highest SP and TC scores on the whole database and all the subsets except for the SP score for the subset RV40. In some cases, MSACompro's improvement was substantial.

**Table 1:** Total SP scores on the full-length BAliBASE 3.0 subsets.

MSA tools	RV11	RV12	RV20	RV30	RV40	RV50
MSACompro	**73.14**	**94.84**	**93.30**	**87.16**	92.11	**91.41**
Clustalw	50.06	86.44	85.16	69.76	78.93	74.24
DIALIGN-TX	51.52	89.18	87.87	73.64	83.64	82.28
FSA	50.28	92.38	86.7	66.27	85.87	78.21
MAFFT	55.13	88.82	89.33	79.08	87.55	84.69
MSAProbs	68.18	94.65	92.81	83.19	**92.47**	90.76
MUSCLE	57.16	91.54	88.91	78.24	86.49	83.52
Opal	66.18	93.70	90.39	80.18	76.25	87.36
POA	37.96	83.19	85.28	69.18	78.22	71.49
Probalign	69.51	94.64	92.57	82.03	92.19	88.86
ProbCons	66.97	94.12	91.67	81.28	90.34	89.41
T-coffee	66.77	94.08	91.61	80.57	89.96	89.43

Bold denotes the highest scores. MSACompro yielded the highest SP scores on all the subsets except RV40. On some datasets such as RV11 and RV30, the improvement is substantial.

**Table 2:** Total TC scores on the full-length BAliBASE 3.0 subsets.

MSA tools	RV11	RV12	RV20	RV30	RV40	RV50
MSACompro	**47.13**	**86.93**	**47.16**	**58.63**	**64.42**	**63.43**
Clustalw	22.74	71.30	21.98	25.63	39.55	30.75
DIALIGN-TX	26.53	75.23	30.49	36.83	44.82	46.56
FSA	26.95	81.77	18.68	24.63	47.43	39.81
MAFFT	28.05	74.36	32.85	41.07	47.51	49.31
MSAProbs	44.11	86.5	46.44	57.63	62.18	60.75
MUSCLE	31.79	80.39	35	38.6	45.02	45.94
Opal	41.97	84.05	34.61	42.03	51.35	50.06
POA	15.26	63.84	23.34	26.73	33.67	27
Probalign	45.34	86.20	43.93	53.6	60.31	54.94
ProbCons	41.66	85.55	40.63	51.47	53.22	57.31
T-coffee	42.29	85.25	38.88	47	55.94	58.69

Bold denotes the highest scores. MSACompro yielded the highest TC scores on all the subsets.

**Table 3:** Overall mean SP and TC scores on the full-length BAliBASE 3.0 subsets.

MSA tools	Mean SP score	Mean TC score
MSACompro	**88.846**	**61.313**
Clustalw	74.980	37.161
DIALIGN-TX	78.48	44.10
FSA	77.878	41.688
MAFFT	81.112	46.028
MSAProbs	87.336	60.248
MUSCLE	81.496	47.151
Opal	82.030	51.789
POA	71.795	33.165
Probalign	87.161	58.528
ProbCons	85.965	55.422
T-coffee	85.728	55.239

Bold denotes the highest scores. MSACompro has the highest mean SP and TC scores.

**Figure 1:**
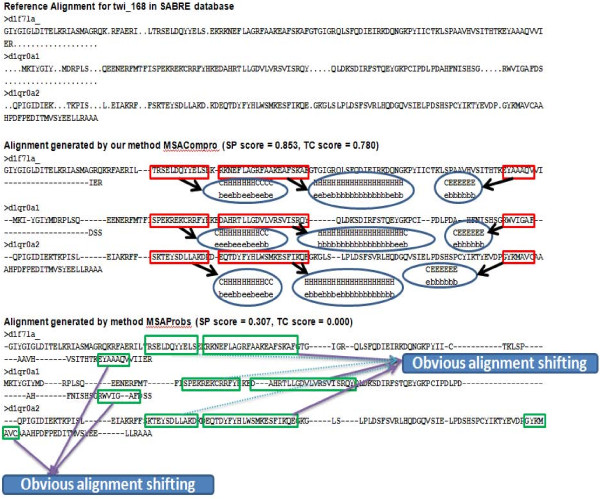
**an example in SABRE database comparing the alignments generated by our method and MSAProbs**. The reference alignment and resulting alignments generated by both methods are respectively shown in the figure. The correct alignment regions significantly improved by our MSACompro after taking structural information are marked in red rectangles. In contrast, the corresponding incorrect alignment regions generated by MSAProbs are represented in green rectangles. The predicted secondary structure and solvent accessibility information for the correctly aligned regions are shown in circles.

Secondly, we evaluated MSACompro and other tools without the help of structural information on the SABmark database [4], which is a very challenging data set for multiple sequence alignment according to a comprehensive study [50]. SABmark is an automatically generated data set consisting of two sets. One set is from SOFI [51] and the other is from the ASTRAL database [52], which contains remote homologous sequences in twilight-zone or superfamily. Since some pairwise reference alignments in SABmark are not generally consistent with multiple alignments, a subset of SABmark, 1.65 called SABRE [53], has been widely used as a multiple sequence alignment benchmark database. SABRE was constructed by identifying mutually consistent columns (MCCs) in the pairwise reference structure alignment. MCCs are considered similar to BAliBASE core blocks. SABRE contains 423 out of 634 SABmark groups that have eight or more MCCs. Table 4 shows the overall mean SP and TC scores of the alignments. The mean SP and TC scores of MSACompro are 8.3 and 9.1 points higher than those of the second best-performer, MSAProbs, demonstrating that incorporating predicted structural features into multiple sequence alignments can substantially improve alignment accuracy for even remotely related homologous sequences. Figure 1 shows an example comparison between the alignments generated by our method, MSACompro, and MSAProbs from the SABRE database. The SP and TC scores significantly improved from 0.307 to 0.853 and 0 to 0.780, respectively. This case demonstrates that taking predicted structural information can help avert aligning unmatched regions, especially when the sequence similarity is unrecognizable.

**Table 4:** Overall mean SP and TC scores on the SABmark 1.65.

MSA tools	Mean SP score	Mean TC score
MSACompro	**68.85**	**49.07**
Clustalw	52.18	31.17
DIALIGN-TX	50.49	29.66
FSA	46.03	25.73
MAFFT	51.99	31.72
MSAProbs	60.55	39.95
MUSCLE	54.99	34.35
Opal	58.28	37.84
POA	38.28	19.02
Probalign	59.96	38.66
ProbCons	59.81	38.99
T-coffee	59.49	39.08

Bold denotes the highest scores. The improvement of SP and TC scores on this data set is substantial.

Thirdly, we also assessed all the tools without using the structural information on the OXBENCH database [54]. OXBENCH is also a popular benchmark database generated by the AMPS multiple alignment method from the 3Dee database of protein structural domains [55]. The conserved columns in OXBENCH can be considered similar to BAliBASE core blocks. The mean SP and TC scores over the whole database are shown in Table 5. The results show that MSACompro improves the alignment accuracy over all other methods.

**Table 5:** Overall mean SP and TC scores on the OXBENCH. Bold denotes highest scores.

MSA tools	Mean SP score	Mean TC score
MSACompro	**92.60**	**84.99**
Clustalw	89.45	80.19
DIALIGN-TX	86.25	75.29
FSA	86.47	75.79
MAFFT	87.58	76.75
MSAProbs	90.06	81.40
MUSCLE	89.50	80.34
Opal	89.38	79.77
POA	82.19	68.40
Probalign	89.97	81.39
ProbCons	89.68	80.52
T-coffee	89.56	80.27

Finally, we also compared the SP scores and TC scores of MSACompro and other tools which adopt the structural information on the six subsets of BAliBASE database, SABmark database and OXBENCH database. Tables 6 and 7 demonstrate the SP and TC scores across the three databases. The results show that MSACompro gained the highest scores on three out of six subsets of BAliBASE and achieved the third highest scores on other data sets, which are lower than PROMALS3D that used true experimental structures as input and PROMALS that used both predicted secondary structures and additional homologous protein sequences found by PSI-BLAST search's on a large protein sequence database [15]. Overall, MSACompro performed similarly as PROMALS, whereas the latter has an advantage on a remote homologous protein sequence data set SABmark since it directly incorporates additional homologous protein sequences to improve the alignment of remotely related target sequences during the progressive alignment process. Moreover, the accuracy of MSACompro on the BAliBASE 3.0 data sets seems to be higher than the published results of another alignment tool of using secondary structure information - DIALIGN-SEC [12], which was not directly tested in our experiment because it is only available as a web server other than a downloadable software package. Therefore, MSACompro is useful to improve the accuracy of multiple sequence alignment in general and particularly for most cases in reality where experimental structures are not available.

**Table 6:** Total SP scores of the tools which use the structural information on BAliBASE 3.0 subsets, SABmark data sets and OXBENCH data sets.

MSA tools	RV11	RV12	RV20	RV30	RV40	RV50	Whole BAliBASE	SABmark	OXBENCH
MSACompro	73.14	**94.84**	93.30	87.16	**92.11**	**91.41**	88.85	68.85	92.60
MUMMALS	66.94	94.30	91.04	84.79	87.15	87.91	85.53	62.12	90.25
PROMALS	79.08	93.55	93.31	88.30	89.80	90.27	89.00	77.40	93.76
PROMALS3D	**83.58**	92.33	**93.62**	**89.42**	90.93	89.73	**90.14**	**88.89**	**97.37**

Bold denotes the highest scores.

**Table 7:** Total TC scores of the tools which use the structural information on BAliBASE 3.0 subsets, SABmark data sets and OXBENCH data sets.

MSA tools	RV11	RV12	RV20	RV30	RV40	RV50	Whole BAliBASE	SABmark	OXBENCH
MSACompro	47.13	**86.93**	47.16	58.63	**64.42**	**63.43**	61.31	49.07	84.99
MUMMALS	41.61	83.98	42.83	49.40	48.55	52.88	53.85	41.96	81.43
PROMALS	58.24	81.73	49.59	51.63	50.84	57.19	59.27	60.95	86.73
PROMALS3D	**66.71**	79.30	**55.95**	**61.07**	51.67	54.38	**62.16**	**80.22**	**93.25**

Bold denotes the highest scores.

In order to check if alignment score differences between MSACompro and the other alignment methods are statistically significant, we carried out the Wilcoxon matched-pair signed-rank test [56] on both SP and TC scores of these methods on the three data sets. The p-values of alignment score differences calculated by the Wilcoxon matched-pair signed-rank test are reported in Table 8. Generally speaking, the alignment scores of MSACompro are significantly higher than all the alignment methods without using structural information and MUMMALS of using structural information in all but one case according to the significance threshold of 0.05. The exception is that MSACompro's TC score is higher than MSAProbs on the BAliBASE, but not statistically significant. However, the alignment scores of MSACompro are mostly statistically lower than the other two alignment methods (PROMALS or PROMALS3D) of using predicted structural features, more homologous sequences, or tertiary structures.

**Table 8:** The statistical significance (i.e. p-values) of SP and TC alignment score differences between MSACompro and the other tools on three benchmark data sets.

MSA tools/Score Type	Whole BAliBASE	SABmark	OXBENCH
Clustalw/SP score	< 2.2 × 10^-16^	< 2.2 × 10^-16^	< 2.2 × 10^-16^
Clustalw/TC score	< 2.2 × 10^-16^	< 2.2 × 10^-16^	< 2.2 × 10^-16^
DIALIGN-TX/SP score	< 2.2 × 10^-16^	< 2.2 × 10^-16^	< 2.2 × 10^-16^
DIALIGN-TX/TC score	< 2.2 × 10^-16^	< 2.2 × 10^-16^	< 2.2 × 10^-16^
FSA/SP score	< 2.2 × 10^-16^	< 2.2 × 10^-16^	< 2.2 × 10^-16^
FSA/TC score	< 2.2 × 10^-16^	< 2.2 × 10^-16^	< 2.2 × 10^-16^
MAFFT/SP score	< 2.2 × 10^-16^	< 2.2 × 10^-16^	< 2.2 × 10^-16^
MAFFT/TC score	< 2.2 × 10^-16^	< 2.2 × 10^-16^	< 2.2 × 10^-16^
MSAProbs/SP score	2.931 × 10^-3^	< 2.2 × 10^-16^	< 2.2 × 10^-16^
MSAProbs/TC score	0.4839	< 2.2 × 10^-16^	< 2.2 × 10^-16^
MUSCLE/SP score	< 2.2 × 10^-16^	< 2.2 × 10^-16^	< 2.2 × 10^-16^
MUSCLE/TC score	< 2.2 × 10^-16^	< 2.2 × 10^-16^	< 2.2 × 10^-16^
Opal/SP score	3.384 × 10^-16^	< 2.2 × 10^-16^	< 2.2 × 10^-16^
Opal/TC score	2.15 × 10^-14^	< 2.2 × 10^-16^	< 2.2 × 10^-16^
POA/SP score	< 2.2 × 10^-16^	< 2.2 × 10^-16^	< 2.2 × 10^-16^
POA/TC score	< 2.2 × 10^-16^	< 2.2 × 10^-16^	< 2.2 × 10^-16^
Probalign/SP score	2.87 × 10^-6^	< 2.2 × 10^-16^	< 2.2 × 10^-16^
Probalign/TC score	4.158 × 10^-3^	< 2.2 × 10^-16^	< 2.2 × 10^-16^
ProbCons/SP score	2.16 × 10^-15^	< 2.2 × 10^-16^	< 2.2 × 10^-16^
ProbCons/TC score	6.817 × 10^-7^	< 2.2 × 10^-16^	< 2.2 × 10^-16^
T-coffee/SP score	1.225 × 10^-14^	< 2.2 × 10^-16^	< 2.2 × 10^-16^
T-coffee/TC score	4.503 × 10^-8^	< 2.2 × 10^-16^	< 2.2 × 10^-16^
MUMMALS/SP score	6.191 × 10^-10^	< 2.2 × 10^-16^	2.446 × 10^-15^
MUMMALS/TC score	8.104 × 10^-5^	< 2.2 × 10^-16^	1.265 × 10^-12^
PROMALS/SP score	0.0116 (-)	< 2.2 × 10^-16 ^(-)	0.0186 (-)
PROMALS/TC score	0.529	< 2.2 × 10^-16 ^(-)	0.0274 (-)
PROMALS3D/SP score	0.0149 (-)	< 2.2 × 10^-16 ^(-)	< 2.2 × 10^-16 ^(-)
PROMALS3D/TC score	0.0078 (-)	< 2.2 × 10^-16 ^(-)	< 2.2 × 10^-16 ^(-)

The p-values were calculated using the Wilcoxon matched-pair signed-rank test. All the p-values except for ones denoted by "(-)" are for hypothesis testing that MSACompro has higher alignment scores than the other methods. The p-values denoted by "(-)" are for hypothesis testing that MSACompro has lower alignment scores than the other methods.

In addition to alignment accuracy, alignment speed is also a factor to consider in time-critical applications. Because it is difficult to rigorously compare the speed of different methods due to the difference in implementation and inputs, we only report the roughly estimated running time of the different methods on BAliBASE based our empirical observations. The fastest methods are ClustalW, MAFFT, MUSCLE, and POA, which used less than one hour. The medium-speed methods that used a few hours to less than one day include FSA, Opal, Probalign, MSAProbs, ProbCons, T-coffee, MUMMALS, and DIALIGN-TX. The more time demanding methods are MSACompro, PROMALS, and PROMALS3D because they need to generate extra information for alignment. We ran both PROMALS and MSACompro on the BAliBASE 3.0 database on an 4 eight-core (i.e. 32 CPU cores) Linux server to calculate their running time. It took about 4 days and 6 hours for PROMALS to run on the whole BAliBASE 3.0 data sets, and about 9 hours and 13 minutes for MSACompro to run on the same data sets. MSACompro was faster because it used a multiple-threading implementation to call SSpro/ACCpro to predict secondary structure and solvent accessibility in parallel. Out of about 9 hours and 13 minutes, about four hours and 17 minutes were used by MSACompro to align sequences if secondary structure and solvent accessibility information was provided. However, if only one CPU core is used, it took around 6 days and 14 hours for SSpro and ACCpro called by MSACompro to predict secondary structure and solvent accessibility information alone, which is time-consuming. Therefore, MSACompro will be slower than PROMALS if it runs a single CPU core, but faster on multiple (> = 3) CPU cores. As for PROMALS3D, it used about 9 days to extract tertiary structure information and make alignments.

### A comprehensive study of the effect of predicted structural information on the alignment accuracy

To understand the impact of predicted secondary structure, relative solvent accessibility, and contact map on the accuracy of multiple sequence alignment, we tested their effects on alignments individually or in combination by adjusting the values of their weights used in the partition function (i.e. for secondary structure and solvent accessibility) or in the distance calculation (i.e. for contact map).

#### I. Effect of secondary structure information

**Figure 2:**
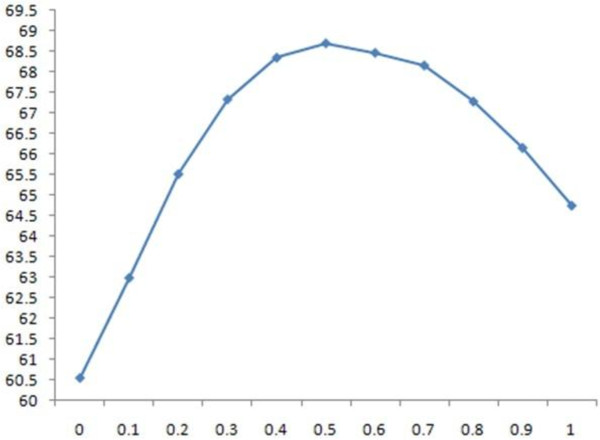
**the 2D plot of SP scores against w_2 _on the SABmark dataset**.

**Figure 3:**
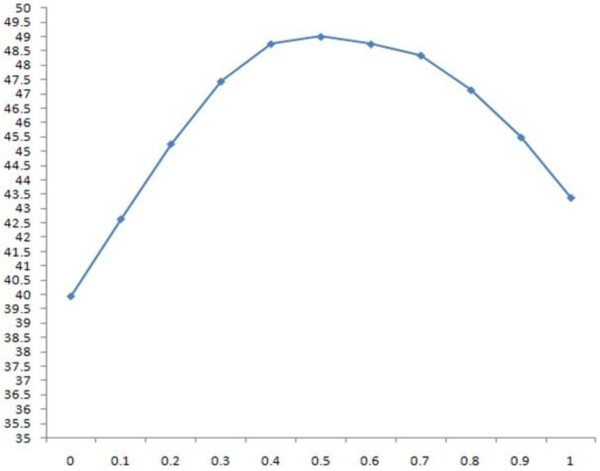
**the 2D plot of TC scores against w_2 _on the SABmark dataset**.

We studied the effect of secondary structure information by adjusting the values of w_1 _(weight for amino acid sequence information) and w_2 _(weight for secondary structure information), the sum of which was kept as 1, and setting the values of w_3 _(weight for relative solvent accessibility) and w_c _(weight for contact map) to 0. The results for different w_2 _values on the SABmark data sets are shown in Table 9. The highest score is denoted in bold and by a superscript of star, and the second highest is denoted in bold. The results show that incorporating secondary structure information always improves alignment accuracy over the baseline established without using secondary structure information (w_2 _= 0). The highest accuracy is achieved when w_2 _is set to .5, at which point the score is 8 points greater than the baseline. w_2 _= 1 means that only secondary structure is used to calculate the posterior alignment probability in the partition function (i.e. equation set (2)), but amino acid sequence similarity is still used to calculate the other posterior alignment probability by the pair Hidden Markov Models. Figures 2 and 3 plot the SP and TC scores against weight values in Table 9 and Table 10, respectively.

**Table 9:** SP scores for different weights of secondary structures on the SABmark benchmark. Bold denotes the two best scores, and an extra superscript of star denotes the highest score.

W_2_	0	0.1	0.2	0.3	0.4	0.5	0.6	0.7	0.8	0.9	1
SP	60.553	62.988	65.514	67.333	68.348	68.698^*^	**68.465**	68.159	67.282	66.153	64.745

The results show that using secondary structure information (i.e. w_2 _> 0) always increases the alignment scores over without using it (i.e. w_2 _= 0). MSACompro yielded the highest accuracy score of ~68.70 when w_2 _is set to 0.5.

**Table 10:** TC scores for different weights of secondary structures on the SABmark benchmark. Bold denotes the two best scores, and an extra superscript of star denotes the highest score.

w_2_	0	0.1	0.2	0.3	0.4	0.5	0.6	0.7	0.8	0.9	1
TC	39.948	42.643	45.262	47.442	48.754	49.005^*^	**48.745**	48.352	47.142	45.4923	43.385

The results show that using secondary structure information (i.e. w_2 _> 0) always increases the alignment scores over without using it (i.e. w_2 _= 0). MSACompro yielded the highest accuracy score of ~68.70 when w_2 _is set to 0.5.

#### II. Effect of relative solvent accessibility information

**Figure 4:**
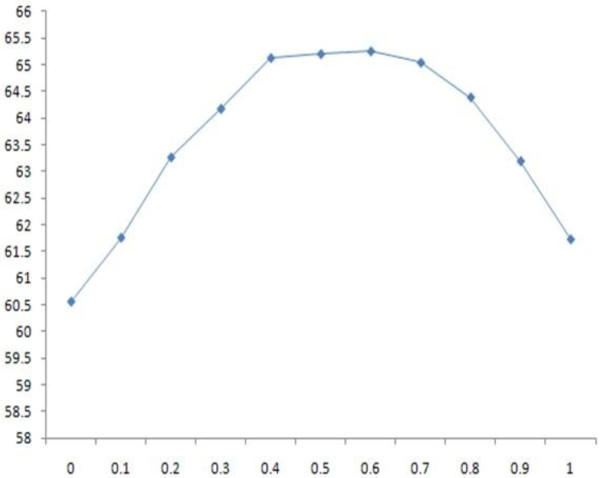
**the 2D plot of SP scores against w_3 _on the SABmark dataset**.

**Figure 5:**
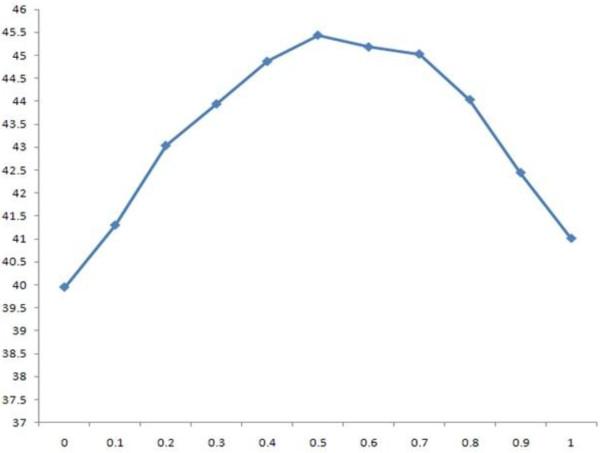
**the 2D plot of TC scores against w3 against the SABmark dataset**.

Similarly, we studied the effect of relative solvent accessibility on the SABmark by adjusting the values of w_1 _and w_3 _and setting the values of w_2 _and w_c _to 0. The SP and TC scores with respect to different weight values are shown in Tables 11 and 12, respectively. The scores are also plotted against the weights in Figures 4 and 5, respectively. The highest SP and TC scores were achieved when w_3 _was set to 0.5 or 0.6.

**Table 11:** SP scores for different weights of relative solvent accessibility on the SABmark benchmark. Bold denotes the two best scores, and an extra superscript of star denotes the highest score.

w_3_	0	0.1	0.2	0.3	0.4	0.5	0.6	0.7	0.8	0.9	1
SP	60.553	61.753	63.260	64.171	65.124	**65.199**	65.249^*^	65.037	64.388	63.1882	61.723

The results show that using relative solvent accessibility information (i.e. w_3 _> 0) always increases the alignment scores over without using it (i.e. w_3 _= 0). MSACompro yielded the highest accuracy score of ~68.70 when w_2 _is set to 0.6.

**Table 12:** TC scores for different weights of relative solvent accessibility on the SABmark benchmark. Bold denotes the two best scores, and an extra superscript of star denotes the highest score.

w_3_	0	0.1	0.2	0.3	0.4	0.5	0.6	0.7	0.8	0.9	1
TC	39.948	41.300	43.035	43.943	44.870	45.442^*^	**45.184**	45.031	44.0383	42.4471	41.012

The results show that using relative solvent accessibility information (i.e. w_3 _> 0) always increases the alignment scores over without using it (i.e. w_3 _= 0). MSACompro yielded the highest accuracy score of ~68.70 when w_2 _is set to 0.5.

#### III. Effect of residue-residue contact map information

We investigated the effect of contact map information on the BAliBASE 3.0 data set by adjusting w_c _and setting w_2 _and w_3 _to 0. We used NNcon to successfully predict the contact maps for subset RV11, RV30, 42 out of 44 alignments in RV12, 38 out of 40 in RV20, 33 out of 46 in RV40, and 14 out of 16 in RV50. We tested the MSACompro method against this data with contact predictions. Tables 13 and 14 show the SP and TC scores for different w_c _values on the subsets of the BAliBASE dataset. The results show that using contact information improved the alignment accuracy on some, but not all, subsets.

**Table 13:** SP scores for different weights for contact map on the BAliBASE3.0 database. Red color highlights the improved scores on each BAliBASE subset. Bold denotes the increased scores.

subset\w_c _wc	0	0.1	0.2	0.3	0.4	0.5	0.6	0.7	0.8	0.9	1
RV11	0.6829	**0.686**	**0.686**	**0.684**	**0.684**	**0.683**	**0.687**	**0.684**	**0.687**	**0.687**	0.668

RV12	0.9461	0.946	0.946	0.945	0.946	0.945	0.946	0.945	0.946	0.945	0.944

RV20	0.9297	0.927	0.926	0.926	0.926	0.926	0.926	0.926	0.926	0.927	0.924

RV30	0.865	0.865	0.864	0.864	0.864	0.863	0.863	0.864	0.864	**0.865**	0.817

RV40	0.928	0.926	0.926	0.924	0.923	0.924	0.924	**0.936**	**0.934**	**0.933**	0.927

RV50	0.9091	0.908	**0.910**	**0.910**	**0.909**	**0.909**	**0.909**	0.907	0.907	0.908	0.886

**Table 14:** TC scores for different weights for contact map on the BAliBASE 3.0 database. Red highlights the improved scores on each BAliBASE subset. Bold denotes the increased scores.

subset\w_c _wc	0	0.1	0.2	0.3	0.4	0.5	0.6	0.7	0.8	0.9	1
RV11	0.441	**0.445**	**0.445**	**0.444**	**0.444**	**0.444**	**0.447**	**0.447**	**0.448**	**0.451**	0.417

RV12	0.8669	0.865	0.866	0.866	**0.866**	0.866	**0.867**	**0.867**	**0.867**	0.865	0.858

RV20	0.482	0.479	0.473	0.460	0.457	0.462	0.453	0.453	0.457	0.453	0.419

RV30	0.607	0.605	0.594	0.594	0.592	0.592	0.591	0.591	0.593	0.592	0.415

RV40	0.67	0.667	0.667	0.661	0.659	0.662	0.662	**0.682**	**0.682**	**0.681**	0.642

RV50	0.625	0.621	**0.634**	**0.633**	**0.629**	**0.628**	**0.631**	0.615	0.615	0.603	0.556

#### IV. Effect of combining secondary structure and solvent accessibility information

**Figure 6:**
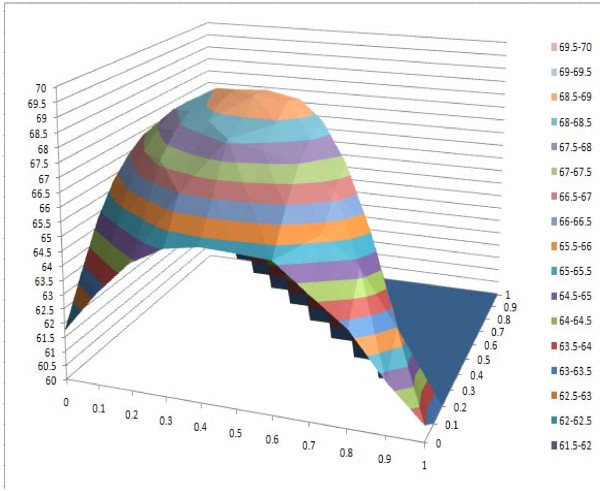
**3D plot of SP scores against secondary structure weight w_2 _and relative solvent accessibility weight w_3_**.

**Figure 7:**
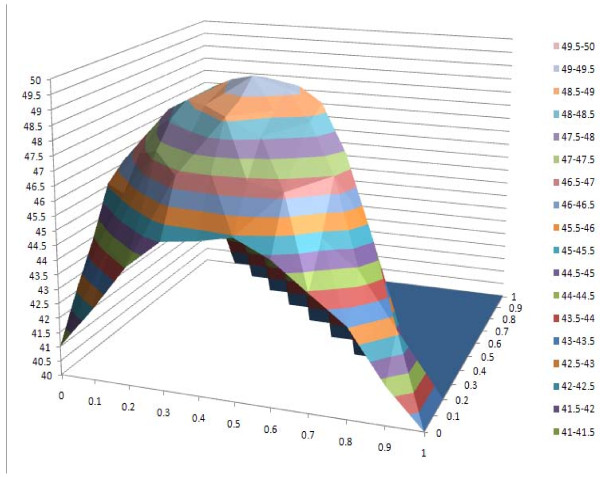
**3D plot of TC scores against secondary structure weight w_2 _and relative solvent accessibility weight w_3_**.

We adjusted the values of w1 (weight for amino acid), w2 (weight for secondary structure) and w3 (weight for relative solvent accessibility) simultaneously to investigate the effect of using secondary structure and relative solvent accessibility together. SP and TC scores on different parameter combinations are shown in Tables 15 and 15. The highest score is denoted in bold and by a superscript of 1, the second in bold and by a superscript of 2, and the third in bold and by a superscript of 3. The results show that the highest scores are achieved when w1 ranges from 0.4 to 0.5, w2 from 0.4 to 0.5, and w3 from 0.1 to 0.2. Also, using both secondary structure and solvent accessibility improves alignment accuracy over using either one. The best alignment score, which uses both secondary structure and solvent accessibility, is >8 points higher than the baseline approach, which does not use them. The changes of SP scores and TC scores with respect to the weights are visualized by the 3D plots in Figures 6 and 7. We conducted similar experiments on BAliBASE 3.0 and OXBENCH and got the similar results (data not shown).

**Table 15:** SP scores for different weight combinations (w_1 _- amino acid, w_2 _- secondary structure, w_3 _- solvent accessibility) on the SABmark 1.65 dataset.

w_2_\w_1_	0	0.1	0.2	0.3	0.4	0.5	0.6	0.7	0.8	0.9	1
0	61.723	63.188	64.388	65.037	65.249	65.199	65.124	64.171	63.260	61.753	60.553

0.1	63.303	64.600	65.635	66.492	66.702	66.619	66.423	65.717	64.790	62.988	

0.2	64.759	66.055	67.161	67.598	68.104	67.831	67.469	66.775	65.514		

0.3	65.781	66.974	67.867	68.312	68.414	68.418	68.033	67.333			

0.4	66.424	67.531	68.251	68.743	**69.016**^**1**^	**68.920**^**2**^	68.3475				

0.5	66.847	67.907	68.4	68.859	**68.933**^**3**^	68.698					

0.6	66.843	67.911	68.544	68.560	68.465						

0.7	66.739	67.800	68.135	68.159							

0.8	66.389	67.119	67.282								

0.9	65.445	66.153									

1	64.745										

Bold denotes the top 3 highest scores. The highest score is indicated by a superscript of 1, the second highest by a superscript of 2, and the third highest by a superscript of 3. The table only shows the values of w_1 _and w_2 _because w_3 _can be inferred by 1 - w_1 _- w_2_.

**Table 16:** TC scores scores for different weight combinations (w_1 _- amino acid, w_2 _- secondary structure, w_3 _- solvent accessibility) on the SABmark 1.65 dataset.

w_2_\w_1_	0	0.1	0.2	0.3	0.4	0.5	0.6	0.7	0.8	0.9	1
0	41.012	42.447	44.038	45.031	45.184	45.442	44.870	43.943	43.035	41.300	39.948

0.1	42.558	44.147	45.596	46.863	47.043	46.910	46.676	45.333	44.390	42.643	

0.2	43.915	45.678	47.270	47.927	48.619	48.080	47.584	47.002	45.262		

0.3	45.582	46.768	48.116	48.660	48.905	48.660	48.371	47.442			

0.4	46.104	47.340	48.473	48.889	**49.508**^**1**^	**49.1589**^**2**^	48.754				

0.5	46.440	47.809	48.210	49.078	**49.222**^**3**^	49.005					

0.6	46.577	47.619	48.487	48.797	48.745						

0.7	46.147	47.579	48.083	48.352							

0.8	45.714	46.898	47.142								

0.9	44.442	45.492									

1	43.385										

Bold denotes the top 3 highest scores. The highest score is indicated by a superscript of 1, the second highest by a superscript of 2, and the third by a superscript of 3. The table only shows the values of w_1 _and w_2 _because w_3 _can be inferred by 1 - w_1 _- w_2_.

#### V. Effect of using contact map information together with secondary structure and solvent accessibility information

In order to study whether or not contact information can be used effectively with secondary structure and solvent accessibility, we adjusted the weight w_c _for contact information, while keeping the w1, w2, and w3 at their optimum values (0.4, 0.5, and 0.1 respectively). Tables 17 and 18 report the SP and TC scores on the BAliBASE 3.0 data set for different w_c _values from no contact information (w_c _= 0) to maximum contact information (w_c _= 1). The results show that the improvement caused by contact information seems not to be substantial and significant.

**Table 17:** SP scores for different contact map weight w_c _on the BAliBASE3.0 database while keeping the weights for amino acid, secondary structure, solvent accessibility to 0.4, 0.5, and 0.1, respectively.

subset\the weight w_c_	0	0.1	0.2	0.3	0.4	0.5	0.6	0.7	0.8	0.9	1
RV11	0.729	**0.730**	0.728	0.726	0.726	0.726	0.727	0.72547	**0.732**	**0.731**	0.722

RV12	0.947	**0.948**	0.947	**0.949**	**0.948**	**0.948**	**0.948**	**0.94855**	**0.948**	**0.948**	0.945

RV20	0.934	0.933	0.932	**0.934**	0.934	0.934	0.933	0.93282	0.9332	0.933	**0.934**

RV30	0.876	**0.877**	**0.877**	**0.876**	0.873	0.873	0.873	0.87287	0.873	0.872	0.846

RV40	0.909	0.908	**0.909**	**0.909**	**0.909**	**0.909**	**0.909**	**0.909**	0.909	**0.921**	**0.913**

RV50	0.911	0.910	**0.911**	0.909	0.909	0.908	0.902	0.90807	**0.914**	**0.914**	0.871

Bold denotes the increased scores.

**Table 18:** TC scores for different contact map weight w_c _on the BAliBASE3.0 database while keeping the weights for amino acid, secondary structure, solvent accessibility to 0.4, 0.5, and 0.1, respectively.

subset\the weight w_c_	0	0.1	0.2	0.3	0.4	0.5	0.6	0.7	0.8	0.9	1
RV11	0.470	**0.472**	0.471	0.469	0.468	0.468	0.468	0.468	**0.475**	**0.471**	0.450

RV12	0.870	**0.870**	0.869	**0.872**	**0.872**	**0.871**	**0.871**	**0.872**	**0.870**	0.869	0.863

RV20	0.481	0.465	0.460	0.478	0.478	0.477	0.477	0.472	0.471	0.472	0.468

RV30	0.609	0.591	0.590	0.588	0.589	0.588	0.588	0.587	0.589	0.586	0.434

RV40	0.628	0.626	0.624	0.625	0.625	0.625	0.625	0.624	0.6249	**0.644**	0.6124

RV50	0.601	0.595	**0.60071**	**0.601**	0.596	0.596	0.586	**0.625**	**0.63643**	**0.634**	0.55

Bold denotes the increased scores.

## Conclusion

In this work, we designed a new method to incorporate predicted secondary structure, relative solvent accessibility, and residue-residue contact information into multiple protein sequence alignment. Our experiments on three standard benchmarks showed that the method improved multiple sequence alignment accuracy over most existing methods without using secondary structure and solvent accessibility information. However, the performance of the method is comparable to PROMALS and PROMALS3D by slightly lower scores on some subsets and behind it by a large margin on SABMARK probably because these two methods used homologous sequences or tertiary structure information in addition to secondary structure information. Since multiple sequence alignment is often a crucial step for bioinformatics analysis, this new method may help improve the solutions to many bioinformatics problems such as protein sequence analysis, protein structure prediction, protein function prediction, protein interaction analysis, protein mutagenesis and protein engineering.

## Authors' contributions

JC and XD designed the algorithm. XD implemented the algorithm and carried out the experiments. XD and JC analyzed the data. XD and JC wrote the manuscript. XD and JC approved it.
